# Food Diversity and Indigenous Food Systems to Combat Diet-Linked Chronic Diseases

**DOI:** 10.1093/cdn/nzz099

**Published:** 2019-09-02

**Authors:** Dipayan Sarkar, Jacob Walker-Swaney, Kalidas Shetty

**Affiliations:** 1 Department of Plant Sciences; 2 Environmental and Conservation Science Program; 3 Global Institute of Food Security and International Agriculture, North Dakota State University, Fargo, ND, USA

**Keywords:** antidiabetic, plant-based food diversity, Native American ecosystem, noncommunicable chronic diseases, phenolic bioactives, traditional foods, type 2 diabetes

## Abstract

Improving food and nutritional diversity based on the diversity of traditional plant-based foods is an important dietary strategy to address the challenges of rapidly emerging diet- and lifestyle-linked noncommunicable chronic diseases (NCDs) of indigenous communities worldwide. Restoration of native ecosystems, revival of traditional food crop cultivation, and revival of traditional knowledge of food preparation, processing, and preservation are important steps to build dietary support strategies against an NCD epidemic of contemporary indigenous communities. Recent studies have indicated that many traditional plant-based foods of Native Americans provide a rich source of human health–relevant bioactive compounds with diverse health benefits. Based on this rationale of health benefits of traditional plant-based foods, the objective of this review is to present a state-of-the-art comprehensive framework for ecologically and culturally relevant sustainable strategies to restore and integrate the traditional plant food diversity of Native Americans to address the NCD challenges of indigenous and wider nonindigenous communities worldwide.

## Introduction

Loss of plant-based food diversity and subsequent changes in dietary patterns closely associated with higher daily intake of more hyperprocessed and calorie-dense foods have partly contributed to the rapid rise of diet- and lifestyle-linked noncommunicable chronic diseases (NCDs) across different communities worldwide (1, 2). The NCD health challenges linked to reduced traditional plant-based food diversity have been particularly acute for indigenous populations who have witnessed the most significant losses in their traditional food and nonfood resources and have been forced to adapt to nontraditional dietary patterns (3). Globally, modern contemporary diets do offer more diversity in terms of plant-based food sources; however, restricted access and affordability of plant-based foods such as whole grains, legumes, fruits, and vegetables, and their associated health benefits, is a major food and nutritional security challenge for impoverished populations. Such restricted access to healthy food choices along with other socioeconomic burdens, ranging from loss of land, to lack of economic opportunities, to psychological trauma from colonization, have substantially contributed to the worsening NCD-linked health disparities between indigenous and nonindigenous people (4). The occurrence of chronic diseases varies widely among different indigenous tribes; however, NCD-linked reduced life span is prevalent across most indigenous communities worldwide (5, 6).

Most public health reports from last 2 decades have suggested that Native American communities and Alaska Natives of the United States are facing increasing health challenges due to the NCD-linked epidemic of type 2 diabetes (T2D) and cardiovascular diseases (CVDs) (7, 8). A national diabetes statistical report of 2017 indicated that the prevalence of T2D and CVDs among the Native American population is 16.5% higher and average life expectancy is 5.2 y less than other races and ethnic groups in the United States (8). Prevention and management strategies to combat these disease epidemics need a systems approach with a solid foundation in an ecological, cultural, social, and economic framework, and an understanding of the health disparities and solutions from indigenous community perspectives.

The framework and strategies suggested in this review are to revive and revitalize the traditional plant-based food diversity of Native Americans and to integrate such foods into contemporary healthy dietary patterns as part of overall public health solutions for NCD epidemics. Kuhnlein and Receveur (9) defined a “traditional food system” as “all food within a particular culture available from local natural resources and culturally acceptable.” Such culturally acceptable food from specific ecological origins largely shaped the traditional dietary patterns of most indigenous communities. Over centuries, new foods have been introduced or integrated into these traditional food systems by settlers and immigrants, which led to the development of new dietary patterns worldwide. Therefore, traditional plant-based foods of Native Americans, especially the diversity of heirloom cultivars of colored corn, climbing bean, squash, root crops, and native berries, with a high human health-relevant bioactive profile, can be incorporated into a contemporary diet by integrating with other nontraditional healthy vegetables, legumes, whole grains, and fruits. This will develop dietary support strategies targeting NCD-linked health benefits for indigenous and wider nonindigenous communities (10–12). Additionally, these traditional food plants and their domesticated and wild races are more resilient in native ecosystems and therefore potentially more resilient to projected changes in climate due to global warming (13). Therefore, targeting these traditional food plants based on their evolutionary advantages and diversity to address resiliency to a rapid rise in climate change–linked food insecurity and public health challenges such as the NCD epidemic in the Native American population has significant relevance (12, 13). The evolution and domestication of these traditional food plants in native communities with specific climate and soil niches has a sound scientific foundation and needs special attention to develop a healthy dietary pattern to counter NCD-linked health disparities of Native American communities (12). Such a sustainable dietary strategy of incorporating traditional food plants of Native Americans can help to build a more resilient plant-based food system and can be targeted toward widening and improving the potential food diversity–linked benefits for NCDs. Therefore, the objective of this review is to present a state-of-the-art ecological and cultural perspective of traditional food systems of Native Americans, and to advance sustainable and community-oriented strategies for restoring and integrating traditional plant-based foods in contemporary diets to address NCD-linked public health challenges of indigenous and wider nonindigenous communities. To compile this review, Google Scholar and Science Direct web search engines were used, and relevant scientific research (in vitro and epidemiological) published mostly after 2000 was included. A few fundamental studies of traditional food systems and public health challenges of Native American populations published between 1995 and 2000 were also included for a better understanding of the impact of changing dietary patterns during the 20th century on NCD-linked health disparities of contemporary Native American communities.

## Ecological Foundations of Food, Nutrition, and Health of Indigenous Communities in the Americas

The foundations of life, such as food resources, medicines, cultures, rituals, and spiritual integrity, of indigenous Native American communities have long been rooted and associated with their respective native and localized ecosystems (14). Diverse native ecosystems of this continent from tundra to prairie grassland to woodland to coastal ecosystems have provided the basic foundations of life for all Native American tribes since their earliest settlements and shaped the social and cultural fabric of these indigenous communities (15). However, most of the original and native ecosystems of this continent have been altered or degraded, especially rapidly in the last 2 centuries, due to severe anthropogenic alterations and associated disturbances (16). For centuries, Native American tribes employed long-developed and well-tested traditional conservation practices such as use of controlled fire regimes for revitalization of native flora, natural grazing, grazing pastures in rotations, judicious harvesting of plant and animal food resources, reliance on the natural cycle of seasons to harvest foods and allowing the natural food resources to recycle, bioconservation of native aquatic life, and overall preservation and protection of native ecosystems (17, 18). In this context, indigenous tribes’ traditional knowledge of their respective ecosystems long guided these communities to judiciously use ecological resources with balanced and sustainable approaches, and to maintain the original characteristics of native ecosystems by adopting different renewable strategies (19, 20). Domestication and cultivation of traditional food plants with enhanced resilience in the native ecosystem was also part of such traditional knowledge and wisdom.

Therefore, a resilient native ecosystem with high species biodiversity, including plant-based food diversity, is essential for the sustainability and well-being of dislocated Native American communities (20). The negative impacts of the loss of native ecosystems will become more complex in the near future with increasing challenges linked to climate change and its coupled pressure on the environment, food production, and public health (21, 22). It is therefore important to revive traditional knowledge to revitalize native ecosystems, specifically to enhance diversity of traditional plant foods and their subsequent integration in contemporary diets. This is essential to build the overall resilience of Native American communities based on a sustainable dietary framework (22). Such a revival of traditional food plant diversity based on sound ecological and cultural foundations will help to improve the health-targeted bioactive richness of overall nutritional composition of contemporary diets of Native American communities, which is especially critical for addressing NCD-linked health disparities.

## Loss of Food Diversity: Impacts on Health and Well-Being of Indigenous Native American Communities

Prior to developing sound food diversity and nutritional intervention strategies to advance public health policies addressing NCD-linked health disparities of Native American communities, it is important to understand and recognize significant dietary and lifestyle changes and what has happened in the socioeconomic and ecological domains in these communities. Until the early 20th century, most Native American tribes farmed, hunted, or fished on their own lands and wetlands or gathered foods and medicinal plants from the woods and forests (14). The traditional Native American diet comprised mostly low-fat, high-protein, complex carbohydrate-based whole foods with potentially enriched phytochemicals and dietary fiber (9, 14). Such a traditional diet of diverse plant- and animal-based foods combined with high physical activity and relatively low psychological stress maintained a healthy lifestyle.

**FIGURE 1:**
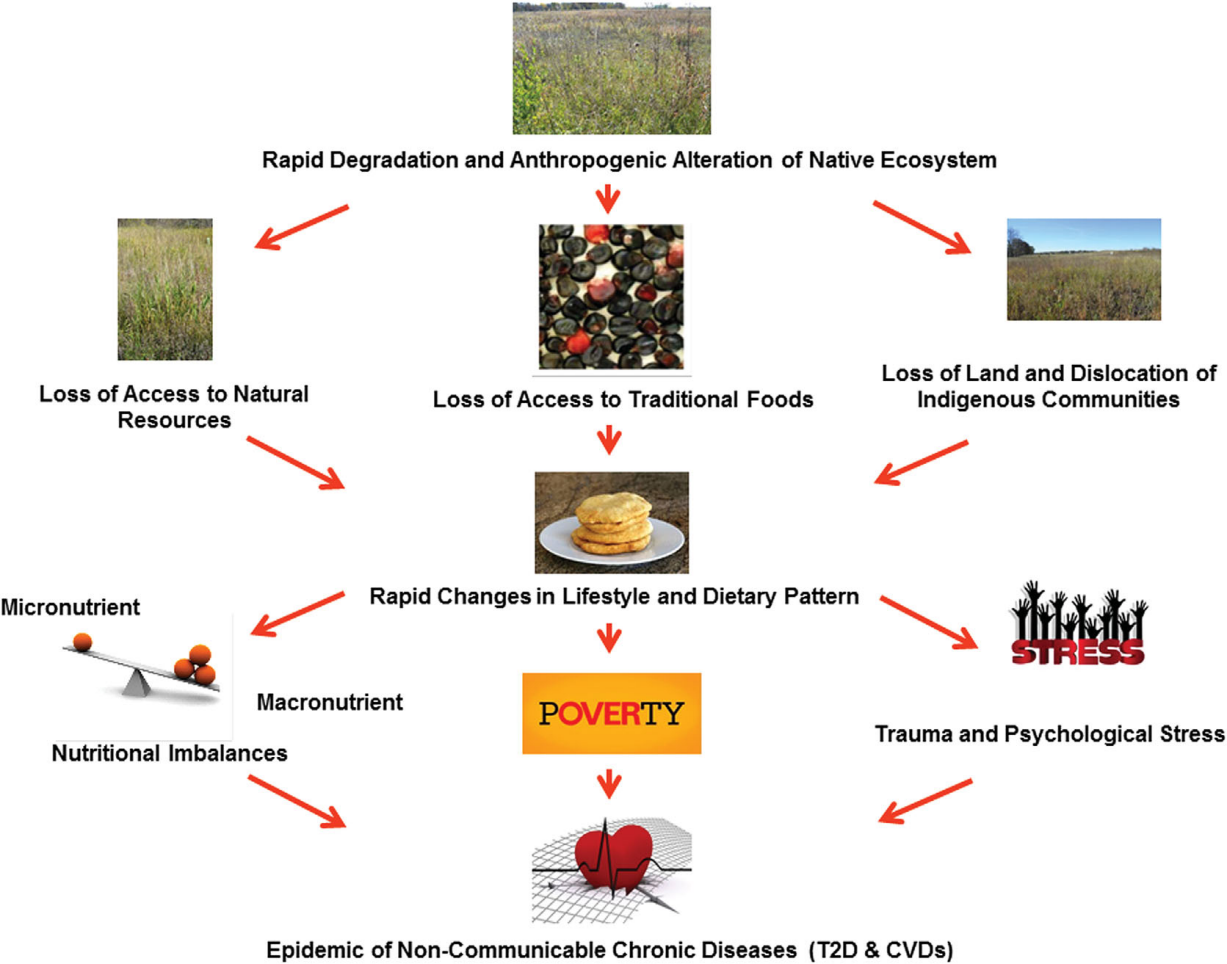
Rapid breakdown of native ecosystem–linked traditional food systems and its impact on chronic disease challenges of Native American communities linked to food and nutritional insecurity. CVD, cardiovascular disease; T2D, type 2 diabetes.

However, in the last century the growth of industry and technology triggered a general shift from home cooking to higher consumption of commercially produced food products from a few calorie-dense food plants and rise in commercial animal farming that led to the development of multiple popular food chains across the United States (23). To support the demands of this new food industry, plant breeding strategies were also focused on developing new varieties of major food plants with higher yield-linked macronutrient profiles while neglecting other essential and beneficial nutritional components of food important for good health (24). Furthermore, beneficial nonmacronutrient components of major foods such as micronutrients, vitamins, phytochemicals, and fiber, were also removed during postharvest food processing. Additionally, industrialized monocropping with limited food plant choices and their high macronutrient-yielding cultivars gradually replaced traditional food plants and their landraces (25). This replacement of traditional food resources with foods having high concentrations of refined hyperprocessed carbohydrate and fat, besides relocation to reservations, unemployment, and poverty, resulted in a major shift in lifestyle and in dietary patterns of Native American communities (26). Such drastic changes in food habits along with the loss of access to traditional fresh and whole foods partly contributed to the rapid rise of obesity-linked T2D and CVDs (27). Although the cause of NCD-linked health disparities of Native American communities is more complex, involving different socioeconomic, political, and historical factors, the loss of native ecosystems and subsequent loss in traditional food plant diversity and related rapid changes in dietary patterns had a profound negative impact on the overall livelihoods and well-being of these indigenous communities (**Figure 1**).

## Health Disparities in Native American Communities: Focus on NCDs

NCDs such as obesity-linked T2D and CVDs are rapidly increasing in prevalence and becoming the leading cause of death in different communities, including indigenous and wider nonindigenous populations worldwide (3). However, nearly 400 million indigenous people (5% of global population) face significant health disparities compared with their nonindigenous counterparts (28). According to the International Diabetes Federation, >50% of indigenous adults aged >35 y have T2D and many more are at the prediabetic stage (28). In the United States, Native American adults are more (17.3%) likely to have higher diagnosed diabetes rates than non-Hispanic whites (10.1%) (8).

The latest report of the National Center for Chronic Disease Prevention and Health Promotion indicates the highest prevalence of diagnosed diabetes in Native American and Alaska Natives, with 14.9% in men and 15.3% in women (8). However, the prevalence of T2D varies with region and among different Native American tribes, from 6% among Alaska Natives to 22.2% in some Native American tribes of Southwest America (8). In North Dakota, Native Americans have a diabetes prevalence and diabetes mortality rate 2.3 times higher than the non-Hispanic white population, whereas Native American youth aged 10–19 y are 9 times more likely to be diagnosed with T2D (29). Similar to North Dakota, the Native American population of Minnesota also faces a significant health burden from a higher prevalence of T2D (12.3%) than their nonindigenous counterparts (7.8% in the non-Hispanic white population) (30). In addition to T2D and CVDs, gestational diabetes mellitus (GDM) is also becoming a serious public health challenge, with the prevalence in Native American women exceeding that of the non-Hispanic white population. The prevalence rate of GDM in some Native American, Alaska Natives, and Canada First Nation tribes is ≤15% and potentially going to increase in the next couple of decades (31). Because the health of the mother is a major predictor for a child’s health and well-being, even in later stages of adolescence and adulthood, the improvement of maternal health is essential to build healthy future communities. Therefore, public health policies need urgently to address these rapidly growing health challenges of T2D, CVDs, and GDM to reduce the increasing health disparities between Native American communities and nonindigenous races and ethnic groups in the United State and worldwide.

## Traditional Plant-Based Food Diversity of Native Americans

Traditional wisdom, invention, and continuous observation guided early humans to adopt and incorporate plant-based foods in their diet and for medicinal uses depending on their availability and nutritional properties relevant to human well-being (9, 12). Mesoamerica is the center of origin for diverse food plants such as corn, beans, cassava, squash, sweet potato, tomato, pepper, potato, sunflower, papaya, amaranth, quinoa, pineapple, groundnut, and many berries, herbs, and medicinal plants (12, 32). Since around 4000 BC, many of these food plants were domesticated and cultivated by different Native American tribes and were part of their traditional dietary systems (32). Polyculture of colored corn, climbing bean, and squash, widely known as the “three sisters crops,” was a unique intercropping system with diverse agroecological benefits (33–35). Other traditional food plants such as sunflower or tuberous species were also grown together with the “three sisters crops” (34). Due to this wider domestication by various native tribes in different agroecologies, many traditional (heirloom) cultivars of these food plants were also developed over the centuries in specific local ecologies (34). Such diversity in traditional food plants and their diverse cultivars with unique nutritional compositions along with wild edibles and traditional animal-based foods provided the basic nutritional needs of Native American tribes (34, 35). However, not all Native American tribes followed a common agricultural system, as many had developed their own unique food systems based on their local, specific agroecological resources.

In this context, many plant-based foods from very specific and localized ecosystems such as wild rice of the Great Lakes region or cranberry from North-Eastern America were widely used in traditional Native American diets (36, 37). Furthermore, different wild edibles such as chokecherry, juneberry, Jerusalem artichoke, prairie turnip, lamb’s quarter, and wild plum were also used as food sources by different Native American tribes, especially by indigenous people of the Northern Plains (26). There is significant merit in reviving such traditional food plant diversity, especially diverse heirloom cultivars of these species, to develop a more ecologically and culturally relevant and more resilient dietary intervention strategy for the prevention and management of diet- and obesity-linked NCDs. However, it is also important to understand and determine the health-protective nutritional composition and relevant functions of this traditional plant-based food diversity before incorporating such plants in dietary intervention strategies.

## Human Health–Promoting Phytochemicals and Associated Health Benefits of Traditional Food Plants of Native Americans

Several traditional food plants and wild edibles of Mesoamerican origin are rich sources of phytochemicals relevant to human health, such as phenolic bioactives, with diverse health benefits (38–40). These traditional plant-based foods are rich sources of natural antioxidants and can potentially protect animal cells against chronic disease and obesity-induced oxidative damage (38, 39). Beyond their antioxidant potential, the bioactive compounds of many traditional food plants also have other NCD-linked health benefits such as antihyperglycemic, antihypertensive, and antidyslipidemic properties, and microbiome-supporting benefits for gut health (38–40).

Therefore, traditional plant-based foods of Native Americans, with their rich source of natural phenolic antioxidants, can be incorporated in dietary intervention strategies to counter chronic oxidative stress and other metabolic dysfunctions commonly associated with T2D, CVDs, and GDM (26, 38, 40). To support and validate this concept of the health benefits of traditional food plants and their diversity, many studies have been carried out at the laboratory of the corresponding author (KS), and in other research and academic institutions (**Table 1**). Previous and current in vitro studies with “three sisters crops” (native colored corn, squash, and bean) have found strong phenolic-linked antioxidant, antidiabetic, and antihypertensive properties of these traditional food plants (38). Similarly, high phenolic-linked antidiabetic properties were observed in traditional colored corn races of Peruvian origin (41), and in other Andean grains and legumes such as purple corn, quinoa, kaniwa, and tarwi (39).

**TABLE 1:** Phenolic bioactive-linked antioxidant and antihyperglycemic properties of selected traditional food plants of Native Americans and comparisons with contemporary varieties

Food plant		Total soluble phenolic concentration (mg/g)	Total DPPH based antioxidant activity (% inhibition)^1^	In vitro anti-hyperglycemic property relevant α-amylase enzyme inhibitory activity (%)	In vitro anti-hyperglycemic property relevant α-glucosidase enzyme inhibitory activity (%)
Traditional corn	Purple corn (41)	8.0	77	ND	51
	Dark-red corn (38)	0.5	38	32	35
	Oaxacan green corn	1.2	55	34	22
Contemporary corn	Yellow corn	0.2–0.5	10–30	20–50	10–20
Traditional beans	Hidatsa red beans	1.4	45	68	18
	Hopi black bean	2.0	78	82	30
	Algonquin speckled bean	1.8	77	78	8
	Arikara yellow bean	1.6	72	48	27
	Andean legume (41)	4.0	40	ND	20
	Jack bean	1.2	60	50	20
Cotemporary beans	Black bean	1.5	65	30–50	30
	Red kidney bean	1.8	72	50–70	34
Traditional squash	Round orange pumpkin (38)	0.2	33	60	50
	Gete okosomin squash	0.3	10	36	50
Contemporary squash	Butternut	0.3	10	20	10
	Buttercup	0.4	20	35	15
Traditional grains	Wild rice	0.1	25	ND	16
	Quinoa (41)	2.3	86	ND	30
Cotemporary grain	White rice	0.02	5	ND	ND

^1^DPPH, 2,2-Diphenyl-1-picrylhydrazyl; ND, not detected.

Squash is also a rich source of phenolic antioxidants and has natural angiotensin-converting enzyme (ACE) inhibitors, which are important for T2D-linked hypertension management (38). High phenolic-linked antidiabetic and antihypertensive properties were found in traditional gete-okosomin and other squash cultivars (Kaprasob, R., Sarkar, D., Shetty, K. (2017) Unpublished). Purple- and orange-fleshed sweet potato cultivars have also shown high phenolic-linked antidiabetic and antihypertensive properties using in vitro assay models (Chintha, P, Sarkar, D., Shetty, K. 2018 Unpublished). Furthermore, different cultivars of climbing beans that are rich in amino acids, micronutrients, and other human health–relevant phytochemicals can also act effectively as a dietary antidote against T2D, CVDs, and other common diet-linked NCDs (42). Other traditional food plants, such as wild rice with its high fiber content and low glycemic index compared with common white rice cultivars, can be targeted alone or in combination with other plant-based foods to prevent and manage obesity-linked NCDs. Native berries such as chokecherry, juneberry, cranberry, and blueberry are also very high in phenolic bioactives with high antioxidant potential and can be targeted as sources of healthy foods or beverages to improve health outcomes of indigenous communities of the United States and worldwide (43–45). Therefore, all these traditional food plants can be utilized successfully for developing value-added healthy dietary strategies to combat the prevalence of NCDs in indigenous and wider nonindigenous communities experiencing a chronic disease epidemic. However, the dietary intervention strategies must be developed based on systems approaches, especially by emphasizing the ecological and cultural relevance of such strategies to support sustainable food diversity-linked health solutions in indigenous communities.

## Strategies to Enhance Traditional Foods and Nutritional Diversity to Combat the Diet-Linked NCD Epidemic in Native American Communities

**FIGURE 2:**
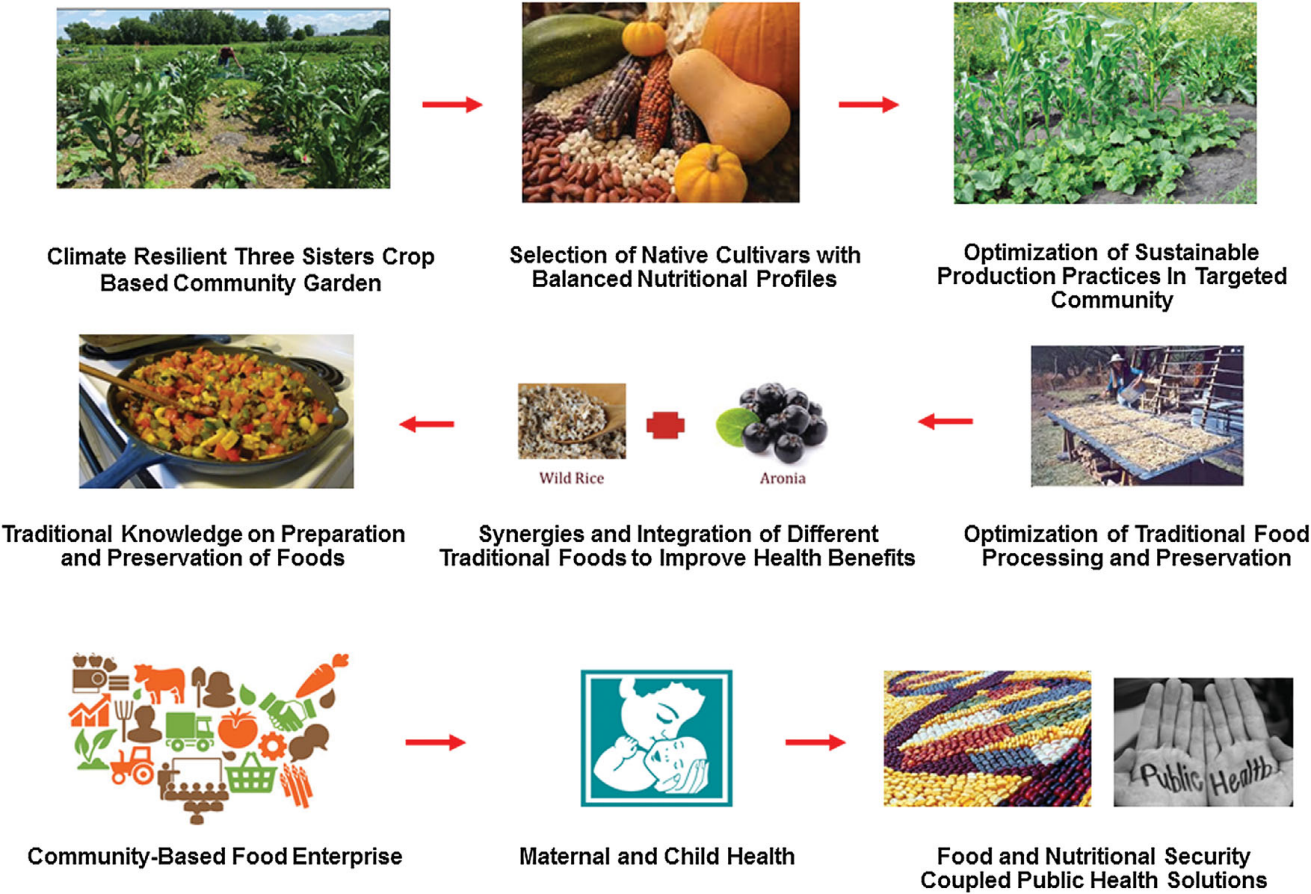
Strategies to enhance traditional food plant diversity to counter the challenges of diet-linked noncommunicable chronic diseases of contemporary Native American and wider nonindigenous communities.

Overall, the average consumption of traditional and ethnic plant-based foods by Native American communities is <10%, and many living in the reservations do not even have access to traditional foods (26). Therefore, there is an urgent need to develop different sustainable strategies and policy measures to enhance traditional food and nutritional diversity by improving access to traditional food plants in Native American communities of the reservations and also indigenous people living outside reservations (46) (**Figure 2**). It is also important to address the issue of affordability, because high rates of poverty mean that many Native Americans cannot afford commercialized traditional foods; these are very expensive, with traditional foods derived from some heirloom cultivars being marketed as high-value specialty foods in just a few grocery outlets (47). Additionally, it is essential to revive traditional knowledge of food processing, preparation, and preservation of traditional and ethnic foods of Native Americans, and to educate young Native Americans about the history and human health benefits of these traditional foods (47). Based on this urgent need to restore traditional plant-based food production and incorporate such foods in contemporary modern diets for wider health benefits, different community-oriented sustainable strategies that can be implemented both in reservation and nonreservation areas are presented. These strategies have been partly implemented in different Native American communities of the Northern Plains as part of the overall food and nutritional security policy goals of the Global Institute of Food Security and International Agriculture at North Dakota State University.

### Documentation and data processing of traditional food plants and other plant-based foods and medicinal resources of different Native American communities of North America

Early human migration and settlement, such as the migration of Native American tribes across North America, was partly guided by the quest for food and based on the availability of diverse plant and animal food resources in their respective ecosystems (19). However, in the last couple of centuries most Native Americans lost their land, witnessed rapid degradation of their native ecosystem, and mostly lost access to traditional food resources. Furthermore, the traditional knowledge of plant-based food diversity of Native Americans was also lost during the last century (26).

Therefore, it is important to revive the knowledge of traditional food systems of Native Americans and restore information about traditional food plants, wild edibles, and other relevant natural food resources (47). To achieve this goal, it is important to engage and involve the elders of indigenous communities to collect and tabulate data and knowledge of traditional foods, especially for building native culture–specific food catalogs and databases. Current scientific advances in computational data mining and data analysis can be used to develop widely accessible data resources on Native American plants and their wider uses in traditional foods and medicines. Such revival of knowledge of traditional plant-based foods of Native Americans and integration of new computational tools will help to restore and build traditional plant-based food dietary systems for diet- and nutrition-linked health benefits of contemporary Native American communities.

### Screening of traditional food plants and their cultivars and other plant-based foods for nutritional profiles and NCD-linked health benefits

Screening and comparing human health–relevant nutritional profiles and diverse health benefits of traditional plant-based foods is essential for building more resilient and robust dietary intervention strategies, especially to counter the diet-linked NCD epidemic of indigenous communities (38, 47). Overall, nutritionally relevant bioactive profiles and associated health benefits of these traditional food plants vary widely between cultivars and wild races, due to different growing conditions and environmental changes, and with different postharvest storage conditions and preservation methods (38–40) (Table 1). Therefore, rapid and metabolically aligned in vitro screening of these traditional food plants and wild edibles for their health-relevant bioactive profiles is important before targeting them in sustainable and culture-specific dietary intervention strategies. Such traditional foods enriched with human health–promoting bioactives will not only help to reduce the NCD-linked health burdens of contemporary Native American communities, but also help wider nonindigenous communities to prevent and manage this NCD epidemic from a wider diversity of food choices from locally adapted ecologies (47, 48). Insights on bioactive profiles and their benefits for human metabolism will also help to target some of these traditional food plants and native medicinal plants for clinically relevant analysis and NCD-related epidemiological studies. Overall, such metabolically driven and robust screening strategies will help to define the optimum health benefits of traditional plant-based foods of Native Americans and to target them for more resilient health outcomes of indigenous and wider nonindigenous communities.

### Reviving traditional knowledge of cultivation of native food plants and conservation methods of native plant species

Revival of traditional knowledge of indigenous culture-specific cultivation practices is as important as understanding the nutritional qualities and associated health benefits of the native food plants. Appreciation of natural food and nonfood resources and their spiritual connection with native ecosystems is a significant part of the ethos of indigenous Native American tribes (49). The traditional cultivation practices of Native American food plants were mostly developed based on continuous observation of the natural life cycle of plants and their connection with the respective environment and ecology. Most Native American tribes also consider the natural resources of their respective ecosystem as part of the same spiritual foundation. Due to such a deep connection with and understanding of the native ecosystem, environmental sustainability and renewal of natural resources were important characteristics of most traditional Native American agricultural practices (50). Therefore, traditional knowledge of different seed preservation and priming methods, design of “three sisters crops” gardens and cultivation practices of this unique polyculture system, timing and method of harvesting of traditional plant-based foods, and different traditional postharvest preservation methods is extremely important. Revival of such traditional knowledge of agriculture and conservation practices and advancing sustainable and resilient traditional food plant systems will also help to reconnect Native American communities with their native ecosystems, which is critical for improving physical and mental health (51).

### Optimization of traditional food processing, food preparation, and food preservation methods to improve NCD-linked health benefits

Similar to different traditional cultivation practices, food preparation, processing, and preservation practices of traditional food plants and wild edibles also have significant relevance in determining optimum nutritional qualities and associated health benefits of traditional Native American foods (52). Another important consideration is that by understanding the cultural context of traditional foods and diet, the targeted indigenous communities can readily participate in the dietary interventions to advance health benefits. In this context, traditional Native American foods such as “succotash,” “wojapi,” “cornbread,” “buffalo stew,” and “wild rice pilaf” can be optimized as human health–relevant nutritionally balanced foods by incorporating bioactive-enriched traditional colored corn, climbing beans, squash, berries, and wild edibles (37). Additionally, native squash, sweet potato, wild rice, and berries rich in natural antioxidants, micronutrients, and dietary fiber can be targeted to design culturally relevant baby foods, especially to address maternal and child nutrition needs in contemporary Native American communities. Such traditional plant-based food dietary strategies can potentially lower the burden of the health care costs and offer more sustainable management options that are readily accessible by and affordable for different age groups of contemporary Native American communities.

### Integration of nonindigenous food plants with superior health benefits in traditional food production and food preparation methods

To address the NCD-linked health disparities of Native American communities, it is also important to integrate nonindigenous food plants and plant-based foods rich in human health–relevant bioactives into traditional Native American diet models (48). Such integration of wider choices of food plants such as nonindigenous vegetables, whole grains, and fruits that are more widely available in the contemporary American diet can help to enhance nutritional diversity and human health–relevant nutritional values of targeted indigenous food models and dietary systems. In our previous study, bioactive-enriched vegetables such as kale, beetroot, and carrot, which are nonindigenous food crops, were integrated into a garden model based on indigenous “three sisters crops” to enhance nutritional diversity and health benefits (48). This previous study found that nonindigenous vegetables such as kale, beetroot, and vegetables with Mesoamerican origins, such as pepper and purple potato rich in phenolic antioxidants and with antihypertensive properties (ACE inhibition), can be integrated in a “three sisters crops” garden model to enhance the food and nutritional diversity of contemporary diets of Native American communities (48). Such a community-based garden strategy with wider food choices and diversity both from traditional and nontraditional food plants can be advanced in Native American reservations and even in urban settings to improve overall access of nutritionally balanced fresh foods to counter diet-linked disease challenges of Native American communities.

### Community engagement and development of socially and culturally relevant food enterprises in Native American communities

Active participation and continuous engagement of Native American communities in decision processes around food and health policy are extremely important for successful outcomes of any intervention strategies (26, 37). Such approaches will not only help to develop more culturally inclusive policy measures but also achieve more comprehensive solutions for the food- and nutrition-linked public health challenges of Native American communities. Additionally, economic empowerment of the indigenous populations of the reservations living in extreme poverty is most critical to address food and nutritional security challenges and to improve the overall health and well-being of these indigenous communities (53). In this context, creating food enterprises and cooperatives in Native American reservations to improve access to and affordability of traditional and nutritionally balanced foods has significant social and economic relevance. Additionally, such approaches will help to improve awareness of traditional and nutritionally balanced foods among youth and future generations of Native American communities, and will guide restoration of their connections with native ecosystems, which has potential for profoundly impacting the physical and psychological health and well-being of indigenous communities.

## Conclusions

Restoring indigenous food sovereignty is essential to address the severe public health challenges linked to food and nutritional security of contemporary Native American communities. However, challenges related to indigenous food sovereignty are extremely complex due to interlinkage of different cultural, ecological, social, political, and economic factors. Therefore, to address such complex indigenous food sovereignty issues, it is important to develop more sustainable and community-oriented strategies based on a systems approach by understanding the ecological, cultural, social, and economic contexts of traditional foods of Native Americans. Additionally, such resilient and robust strategies to improve food and nutritional diversity based on traditional food plants and native medicinal plants of Native Americans should also have enough flexibility to adjust and evolve based on the needs of Native American communities and their contemporary cultural and ecological relevance. Therefore, restoration of traditional food systems of Native Americans and integration of nonindigenous healthy foods from contemporary American diets into traditional food systems to enhance nutritional diversity can have a profound impact on advancing nutritionally linked health outcomes of Native American communities, especially to combat the rapidly increasing NCD epidemic. However, most importantly we have to recognize that access to nutritionally balanced traditional foods is not a privilege but a right of indigenous people of North America and worldwide. Overall, acknowledging and accepting this simple fact can help to develop and advance more effective and sustainable policy measures to enhance traditional food and nutritional diversity in contemporary diets for improving the health, livelihood, and well-being of Native American communities and wider nonindigenous communities.

## Notes

The authors received no specific funding for this work.

Author disclosures: DS, JW-S, and KS, no conflicts of interest.

This article appears as part of the supplement “Proceedings of the Third Annual Conference on Native American Nutrition,” sponsored by the Shakopee Mdewakanton Sioux Community's Seeds of Native Health campaign through a gift to the University of Minnesota. The guest editors of the supplement, Treena Delormier and Mindy S Kurzer, have no conflicts of interest. The opinions expressed in this article are those of the authors and are not attributable to the sponsors or the publisher, Editor, or Editorial Board of *Current Developments in Nutrition*. Publication costs for this supplement were defrayed in part by the payment of page charges.

Abbreviations used: ACE, angiotensin-converting enzyme; CVD, cardiovascular disease; GDM, gestational diabetes mellitus; NCD, noncommunicable chronic disease; T2D, type 2 diabetes.
